# Poly[tetra­aqua­bis­(μ_3_-benzene-1,3-di­carboxyl­ato-κ^3^ *O*:*O*′:*O*′′)bis­(μ_2_-benzene-1,3-dicarboxyl­ato-κ^3^ *O*,*O*′:*O*′′)[μ_2_-1,4-bis­(1,2,4-triazol-1-yl)butane-κ^2^ *N*:*N*′]tetra­zinc(II)]

**DOI:** 10.1107/S1600536810051433

**Published:** 2010-12-15

**Authors:** Gui-Hong Fu

**Affiliations:** a Liaohe Institute of Petroleum Technology, People’s Republic of China

## Abstract

In the crystal structure of the title compound, [Zn_4_(C_8_H_4_O_4_)_4_(C_8_H_12_N_6_)(H_2_O)_4_]_*n*_, one Zn^II^ atom is four-coordinated in a slightly distorted tetra­hedral geometry by two O atoms from benzene-1,3-dicarboxyl­ate (BDC) ligands, one N atom from a 1,4-bis­(1,2,4-triazol-1-yl)butane (BTB) ligand and one water mol­ecule, while a second Zn^II^ atom is five-coordinated in a distorted square-pyramidal geometry bridged by four O atoms from BDC ligands and one water mol­ecule. The Zn^II^ atoms are connected by the benzene-1,3-dicarboxyl­ate anions and the nitro­gen ligand into layers parallel to the *ac* plane. The asymmetric unit consits of two crystallographically independent Zn^II^ cations, two BDC anions and two water mol­ecules in general positions, as well as one-half of the BTB ligand that is completed by inversion symmetry.

## Related literature

For related structures, see: Liu *et al.* (2009 ▶); Wang *et al.* (2009 ▶). 
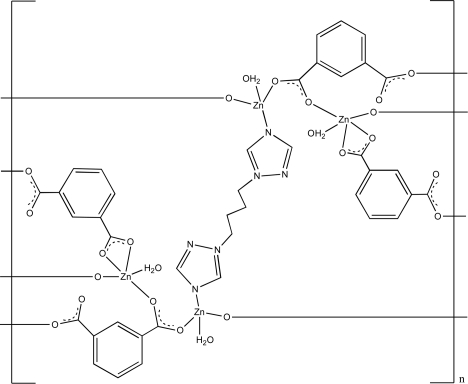


## Experimental

### 

#### Crystal data

[Zn_4_(C_8_H_4_O_4_)_4_(C_8_H_12_N_6_)(H_2_O)_4_]
*M* *_r_* = 1182.23
Monoclinic, 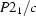

*a* = 10.064 (2) Å
*b* = 21.147 (4) Å
*c* = 10.237 (2) Å
β = 91.76 (3)°
*V* = 2177.7 (7) Å^3^
*Z* = 2
Mo *K*α radiation
μ = 2.27 mm^−1^
*T* = 293 K
0.26 × 0.24 × 0.23 mm

#### Data collection

Bruker APEX CCD area-detector diffractometer
Absorption correction: multi-scan (*SADABS*; Sheldrick, 1996 ▶) *T*_min_ = 0.902, *T*_max_ = 0.918
16853 measured reflections
3819 independent reflections
3381 reflections with *I* > 2σ(*I*)
*R*_int_ = 0.032

#### Refinement

*R*[*F* ^2^ > 2σ(*F* ^2^)] = 0.024
*wR*(*F* ^2^) = 0.058
*S* = 1.04
3819 reflections
316 parameters
H-atom parameters constrained
Δρ_max_ = 0.30 e Å^−3^
Δρ_min_ = −0.25 e Å^−3^

### 

Data collection: *SMART* (Bruker, 2007) ▶; cell refinement: *SAINT* (Bruker, 2007) ▶; data reduction: *SAINT* ▶; program(s) used to solve structure: *SHELXS97* (Sheldrick, 2008 ▶); program(s) used to refine structure: *SHELXL97* (Sheldrick, 2008 ▶); molecular graphics: *SHELXTL-Plus* (Sheldrick, 2008 ▶); software used to prepare material for publication: *SHELXL97*.

## Supplementary Material

Crystal structure: contains datablocks I, global. DOI: 10.1107/S1600536810051433/nc2208sup1.cif

Structure factors: contains datablocks I. DOI: 10.1107/S1600536810051433/nc2208Isup2.hkl

Additional supplementary materials:  crystallographic information; 3D view; checkCIF report

## Figures and Tables

**Fig. 1.**
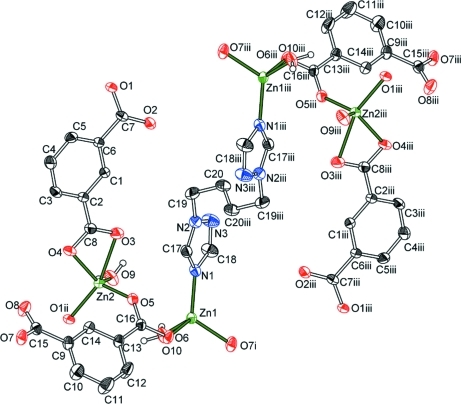
Crystal structure of the title compound with labeling and displacement ellipsoids drawn at the 50% probability level. Symmetry code: i = x,y,z - 1, ii = x - 1, y, z, iii = -x + 1, - y, - z

## supplementary crystallographic information

### Comment

Investigations on metal carboxylate coordination polymers have become of increasing interest in the past few years. As a part of our ongoing investigations in this field (Liu *et al.* 2009; Wang *et al.* 2009), we report here the crystal structure of the title compound. Zn1 ion is surrounded by two oxygen atoms from BDC ligands, one nitrogen atom from BTB ligand and one aqua ligand. Zn2 atom is coordinated to four oxygen atoms from BDC ligands and one aqua ligand (Fig 1). The zinc atoms are linked by the anions into layers parallel to the crystallographic *ac* plane.

### Experimental

The hydrothermal reaction of ZnCl_2_ (0.041 g, 0.3 mmol), 1,4-Bis(1,2,4-triazol-1-yl)butane (btb) (0.078 g, 0.4 mmol) and water (15.0 ml) was carried out at 423 K for 3 d. After cooling to room temperature at a rate of 5 K h^-1^, block-shaped colorless crystals of the title compound suitable for X-ray analysis were obtained.

### Refinement

C-bound H atoms were positioned geometrically (C—H = 0.93 Å) and refined as riding atoms, with Uĩso~(H) = 1.2U~eq~(C). H atoms of the water molecules were initially located in a difference Fourier map, but were idealized and refined as riding atoms, with O—H = 0.85 Å and Uĩso~(H) = 1.5U~eq~(O).

### Crystal data

**Table:**

[Zn_4_(C_8_H_4_O_4_)_4_(C_8_H_12_N_6_)(H_2_O)_4_]	*F*(000) = 1196
*M**_r_* = 1182.23	*D*_x_ = 1.803 Mg m^−^^3^
Monoclinic, *P*2_1_/*c*	Mo *K*α radiation, λ = 0.71073 Å
Hall symbol: -P 2ybc	Cell parameters from 16853 reflections
*a* = 10.064 (2) Å	θ = 3.0–25.0°
*b* = 21.147 (4) Å	µ = 2.27 mm^−^^1^
*c* = 10.237 (2) Å	*T* = 293 K
β = 91.76 (3)°	Block, colourless
*V* = 2177.7 (7) Å^3^	0.26 × 0.24 × 0.23 mm
*Z* = 2	

### Data collection

**Table:**

Bruker APEX CCD area-detector diffractometer	3819 independent reflections
Radiation source: fine-focus sealed tube	3381 reflections with *I* > 2σ(*I*)
graphite	*R*_int_ = 0.032
ω scans	θ_max_ = 25.0°, θ_min_ = 3.0°
Absorption correction: multi-scan (*SADABS*; Sheldrick, 1996)	*h* = −11→11
*T*_min_ = 0.902, *T*_max_ = 0.918	*k* = −25→25
16853 measured reflections	*l* = −12→11

### Refinement

**Table:**

Refinement on *F*^2^	Primary atom site location: structure-invariant direct methods
Least-squares matrix: full	Secondary atom site location: difference Fourier map
*R*[*F*^2^ > 2σ(*F*^2^)] = 0.024	Hydrogen site location: inferred from neighbouring sites
*wR*(*F*^2^) = 0.058	H-atom parameters constrained
*S* = 1.04	*w* = 1/[σ^2^(*F*_o_^2^) + (0.0273*P*)^2^ + 1.178*P*] where *P* = (*F*_o_^2^ + 2*F*_c_^2^)/3
3819 reflections	(Δ/σ)_max_ < 0.001
316 parameters	Δρ_max_ = 0.30 e Å^−^^3^
0 restraints	Δρ_min_ = −0.25 e Å^−^^3^

### Special details

**Table:**

Geometry. All esds (except the esd in the dihedral angle between two l.s. planes) are estimated using the full covariance matrix. The cell esds are taken into account individually in the estimation of esds in distances, angles and torsion angles; correlations between esds in cell parameters are only used when they are defined by crystal symmetry. An approximate (isotropic) treatment of cell esds is used for estimating esds involving l.s. planes.
Refinement. Refinement of F^2^ against ALL reflections. The weighted R-factor wR and goodness of fit S are based on F^2^, conventional R-factors R are based on F, with F set to zero for negative F^2^. The threshold expression of F^2^ > 2sigma(F^2^) is used only for calculating R-factors(gt) etc. and is not relevant to the choice of reflections for refinement. R-factors based on F^2^ are statistically about twice as large as those based on F, and R- factors based on ALL data will be even larger.

### Fractional atomic coordinates and isotropic or equivalent isotropic displacement parameters (Å^2^)

**Table:**

	*x*	*y*	*z*	*U*_iso_*/*U*_eq_	
Zn1	−0.06578 (2)	0.089944 (12)	−0.04961 (2)	0.02203 (8)	
Zn2	0.01752 (2)	0.195761 (12)	0.28205 (2)	0.02292 (8)	
C1	0.4953 (2)	0.19260 (10)	0.3569 (2)	0.0232 (5)	
H1A	0.4831	0.1917	0.2665	0.028*	
C2	0.3852 (2)	0.19388 (11)	0.4356 (2)	0.0253 (5)	
C3	0.4046 (2)	0.19304 (12)	0.5709 (2)	0.0311 (5)	
H3A	0.3318	0.1943	0.6246	0.037*	
C4	0.5317 (2)	0.19028 (12)	0.6257 (2)	0.0341 (6)	
H4A	0.5437	0.1876	0.7159	0.041*	
C5	0.6411 (2)	0.19145 (11)	0.5471 (2)	0.0282 (5)	
H5A	0.7265	0.1914	0.5846	0.034*	
C6	0.6230 (2)	0.19265 (10)	0.4115 (2)	0.0220 (4)	
C7	0.7386 (2)	0.19342 (10)	0.3224 (2)	0.0241 (5)	
C8	0.2511 (2)	0.19557 (11)	0.3714 (2)	0.0277 (5)	
C9	−0.1669 (2)	0.04379 (11)	0.5724 (2)	0.0256 (5)	
C10	−0.2664 (3)	−0.00116 (13)	0.5638 (2)	0.0438 (7)	
H10A	−0.3042	−0.0163	0.6394	0.053*	
C11	−0.3100 (3)	−0.02387 (15)	0.4430 (2)	0.0545 (9)	
H11A	−0.3782	−0.0535	0.4374	0.065*	
C12	−0.2519 (3)	−0.00223 (13)	0.3307 (2)	0.0418 (7)	
H12A	−0.2816	−0.0174	0.2496	0.050*	
C13	−0.1500 (2)	0.04180 (10)	0.3378 (2)	0.0236 (5)	
C14	−0.1084 (2)	0.06503 (10)	0.4595 (2)	0.0229 (5)	
H14A	−0.0408	0.0950	0.4652	0.027*	
C15	−0.1227 (2)	0.07043 (11)	0.7024 (2)	0.0245 (5)	
C16	−0.0893 (2)	0.06554 (10)	0.21533 (19)	0.0210 (5)	
C17	0.2212 (2)	0.08641 (11)	0.0369 (2)	0.0271 (5)	
H17A	0.2034	0.0636	0.1120	0.032*	
C18	0.2031 (3)	0.13749 (13)	−0.1382 (2)	0.0386 (6)	
H18A	0.1651	0.1576	−0.2110	0.046*	
C19	0.4709 (3)	0.08703 (13)	0.0581 (3)	0.0397 (6)	
H19A	0.4611	0.0771	0.1498	0.048*	
H19B	0.5284	0.1237	0.0527	0.048*	
C20	0.5359 (2)	0.03118 (12)	−0.0093 (3)	0.0378 (6)	
H20A	0.5394	0.0400	−0.1021	0.045*	
H20B	0.6266	0.0270	0.0244	0.045*	
N1	0.13007 (18)	0.10764 (9)	−0.04798 (17)	0.0252 (4)	
N2	0.34087 (19)	0.10224 (9)	−0.00052 (18)	0.0295 (4)	
N3	0.3313 (2)	0.13562 (11)	−0.1145 (2)	0.0420 (5)	
O1	0.84932 (14)	0.21495 (8)	0.37012 (15)	0.0288 (4)	
O2	0.72171 (16)	0.17320 (9)	0.20969 (15)	0.0329 (4)	
O3	0.23743 (16)	0.18834 (9)	0.25146 (16)	0.0396 (4)	
O4	0.14763 (15)	0.20414 (9)	0.43934 (16)	0.0367 (4)	
O5	−0.00281 (15)	0.10856 (7)	0.22114 (14)	0.0259 (3)	
O6	−0.12568 (16)	0.04097 (7)	0.10727 (13)	0.0279 (4)	
O7	−0.14620 (17)	0.03846 (8)	0.80488 (13)	0.0305 (4)	
O8	−0.06492 (18)	0.12246 (8)	0.70924 (14)	0.0354 (4)	
O9	0.00911 (17)	0.26150 (8)	0.14493 (15)	0.0359 (4)	
H1	0.0695	0.2706	0.0918	0.054*	
H2	−0.0157	0.2994	0.1567	0.054*	
O10	−0.18152 (16)	0.16534 (8)	−0.02552 (14)	0.0314 (4)	
H3	−0.2116	0.1731	0.0495	0.047*	
H4	−0.1626	0.2010	−0.0583	0.047*	

### Atomic displacement parameters (Å^2^)

**Table:**

	*U*^11^	*U*^22^	*U*^33^	*U*^12^	*U*^13^	*U*^23^
Zn1	0.02386 (14)	0.02685 (15)	0.01542 (13)	−0.00144 (10)	0.00116 (10)	−0.00010 (10)
Zn2	0.01664 (13)	0.02776 (15)	0.02441 (14)	−0.00191 (10)	0.00121 (10)	−0.00385 (10)
C1	0.0200 (11)	0.0293 (12)	0.0204 (10)	−0.0010 (9)	0.0015 (9)	−0.0030 (9)
C2	0.0177 (11)	0.0307 (12)	0.0276 (11)	−0.0024 (9)	0.0035 (9)	−0.0052 (9)
C3	0.0219 (12)	0.0462 (15)	0.0256 (11)	−0.0014 (10)	0.0082 (10)	−0.0025 (10)
C4	0.0321 (14)	0.0491 (16)	0.0214 (11)	−0.0018 (11)	0.0032 (10)	−0.0020 (11)
C5	0.0206 (11)	0.0378 (14)	0.0257 (11)	−0.0010 (10)	−0.0043 (9)	0.0002 (10)
C6	0.0162 (11)	0.0255 (11)	0.0242 (10)	0.0007 (9)	0.0011 (9)	−0.0022 (9)
C7	0.0172 (11)	0.0265 (12)	0.0287 (12)	0.0028 (9)	0.0043 (9)	0.0008 (9)
C8	0.0216 (12)	0.0290 (12)	0.0326 (12)	−0.0034 (9)	0.0037 (10)	−0.0055 (10)
C9	0.0348 (13)	0.0252 (12)	0.0169 (10)	0.0013 (10)	0.0008 (9)	0.0025 (9)
C10	0.0604 (18)	0.0481 (17)	0.0232 (12)	−0.0226 (14)	0.0063 (12)	0.0051 (11)
C11	0.073 (2)	0.061 (2)	0.0303 (14)	−0.0432 (17)	0.0038 (14)	0.0007 (13)
C12	0.0608 (19)	0.0440 (16)	0.0202 (11)	−0.0227 (14)	−0.0031 (12)	−0.0022 (11)
C13	0.0300 (12)	0.0230 (11)	0.0177 (10)	−0.0008 (9)	−0.0003 (9)	0.0008 (8)
C14	0.0271 (12)	0.0202 (11)	0.0212 (10)	−0.0001 (9)	−0.0013 (9)	0.0016 (8)
C15	0.0263 (12)	0.0285 (12)	0.0186 (10)	0.0045 (10)	0.0011 (9)	0.0022 (9)
C16	0.0233 (11)	0.0222 (11)	0.0175 (10)	0.0037 (9)	0.0000 (9)	−0.0008 (8)
C17	0.0276 (12)	0.0309 (13)	0.0229 (11)	0.0015 (10)	0.0036 (10)	−0.0008 (9)
C18	0.0321 (14)	0.0472 (16)	0.0366 (13)	0.0027 (12)	0.0046 (11)	0.0172 (12)
C19	0.0266 (13)	0.0482 (16)	0.0438 (14)	−0.0005 (11)	−0.0074 (11)	−0.0100 (12)
C20	0.0206 (12)	0.0478 (16)	0.0448 (14)	0.0052 (11)	−0.0017 (11)	−0.0019 (12)
N1	0.0263 (10)	0.0276 (10)	0.0219 (9)	0.0026 (8)	0.0041 (8)	0.0005 (8)
N2	0.0267 (11)	0.0316 (11)	0.0303 (10)	0.0020 (8)	0.0010 (8)	−0.0033 (8)
N3	0.0323 (12)	0.0508 (14)	0.0431 (12)	−0.0025 (10)	0.0059 (10)	0.0154 (11)
O1	0.0162 (8)	0.0346 (9)	0.0360 (9)	−0.0024 (7)	0.0061 (7)	−0.0108 (7)
O2	0.0227 (8)	0.0531 (11)	0.0230 (8)	0.0011 (8)	0.0035 (7)	−0.0060 (7)
O3	0.0217 (9)	0.0667 (13)	0.0304 (9)	−0.0031 (8)	−0.0020 (7)	−0.0066 (8)
O4	0.0152 (8)	0.0603 (12)	0.0349 (9)	−0.0010 (8)	0.0037 (7)	−0.0107 (8)
O5	0.0269 (8)	0.0254 (8)	0.0258 (8)	−0.0033 (7)	0.0059 (7)	−0.0051 (6)
O6	0.0372 (9)	0.0319 (9)	0.0144 (7)	−0.0065 (7)	−0.0014 (6)	−0.0011 (6)
O7	0.0453 (10)	0.0317 (9)	0.0146 (7)	−0.0039 (8)	0.0007 (7)	0.0019 (6)
O8	0.0494 (11)	0.0335 (10)	0.0229 (8)	−0.0128 (8)	−0.0026 (7)	−0.0005 (7)
O9	0.0403 (10)	0.0334 (9)	0.0349 (9)	0.0056 (8)	0.0141 (8)	0.0049 (7)
O10	0.0408 (10)	0.0304 (9)	0.0236 (8)	0.0054 (7)	0.0105 (7)	0.0024 (7)

### Geometric parameters (Å, °)

**Table:**

Zn1—O10	1.9943 (16)	C11—C12	1.384 (3)
Zn1—O7^i^	1.9963 (16)	C11—H11A	0.9300
Zn1—N1	2.0058 (19)	C12—C13	1.385 (3)
Zn1—O6	2.0185 (15)	C12—H12A	0.9300
Zn2—O5	1.9553 (16)	C13—C14	1.392 (3)
Zn2—O9	1.9757 (16)	C13—C16	1.498 (3)
Zn2—O1^ii^	1.9840 (15)	C14—H14A	0.9300
Zn2—O4	2.0517 (18)	C15—O8	1.245 (3)
Zn2—O3	2.2503 (17)	C15—O7	1.277 (3)
Zn2—C8	2.496 (2)	C16—O5	1.260 (3)
C1—C6	1.386 (3)	C16—O6	1.266 (2)
C1—C2	1.390 (3)	C17—N2	1.318 (3)
C1—H1A	0.9300	C17—N1	1.322 (3)
C2—C3	1.393 (3)	C17—H17A	0.9300
C2—C8	1.484 (3)	C18—N3	1.306 (3)
C3—C4	1.382 (3)	C18—N1	1.354 (3)
C3—H3A	0.9300	C18—H18A	0.9300
C4—C5	1.384 (3)	C19—N2	1.459 (3)
C4—H4A	0.9300	C19—C20	1.526 (3)
C5—C6	1.395 (3)	C19—H19A	0.9700
C5—H5A	0.9300	C19—H19B	0.9700
C6—C7	1.500 (3)	C20—C20^iii^	1.519 (5)
C7—O2	1.238 (3)	C20—H20A	0.9700
C7—O1	1.286 (3)	C20—H20B	0.9700
C8—O3	1.241 (3)	N2—N3	1.365 (3)
C8—O4	1.282 (3)	O1—Zn2^iv^	1.9840 (15)
C9—C10	1.381 (3)	O7—Zn1^v^	1.9963 (16)
C9—C14	1.388 (3)	O9—H1	0.8500
C9—C15	1.500 (3)	O9—H2	0.8500
C10—C11	1.385 (4)	O10—H3	0.8501
C10—H10A	0.9300	O10—H4	0.8500
			
O10—Zn1—O7^i^	107.66 (7)	C12—C11—H11A	120.1
O10—Zn1—N1	115.30 (7)	C10—C11—H11A	120.1
O7^i^—Zn1—N1	118.93 (7)	C11—C12—C13	120.7 (2)
O10—Zn1—O6	97.01 (7)	C11—C12—H12A	119.7
O7^i^—Zn1—O6	100.94 (6)	C13—C12—H12A	119.7
N1—Zn1—O6	113.98 (7)	C12—C13—C14	119.1 (2)
O5—Zn2—O9	115.80 (7)	C12—C13—C16	120.12 (19)
O5—Zn2—O1^ii^	104.80 (6)	C14—C13—C16	120.8 (2)
O9—Zn2—O1^ii^	99.23 (7)	C9—C14—C13	120.5 (2)
O5—Zn2—O4	112.94 (7)	C9—C14—H14A	119.8
O9—Zn2—O4	120.61 (8)	C13—C14—H14A	119.8
O1^ii^—Zn2—O4	99.13 (7)	O8—C15—O7	121.3 (2)
O5—Zn2—O3	89.06 (7)	O8—C15—C9	120.48 (19)
O9—Zn2—O3	88.34 (7)	O7—C15—C9	118.2 (2)
O1^ii^—Zn2—O3	159.06 (6)	O5—C16—O6	121.23 (18)
O4—Zn2—O3	60.56 (6)	O5—C16—C13	120.12 (18)
O5—Zn2—C8	101.74 (7)	O6—C16—C13	118.64 (19)
O9—Zn2—C8	106.32 (8)	N2—C17—N1	110.2 (2)
O1^ii^—Zn2—C8	129.85 (7)	N2—C17—H17A	124.9
O4—Zn2—C8	30.83 (7)	N1—C17—H17A	124.9
O3—Zn2—C8	29.74 (6)	N3—C18—N1	114.4 (2)
C6—C1—C2	120.77 (19)	N3—C18—H18A	122.8
C6—C1—H1A	119.6	N1—C18—H18A	122.8
C2—C1—H1A	119.6	N2—C19—C20	112.1 (2)
C1—C2—C3	119.1 (2)	N2—C19—H19A	109.2
C1—C2—C8	118.31 (19)	C20—C19—H19A	109.2
C3—C2—C8	122.60 (19)	N2—C19—H19B	109.2
C4—C3—C2	120.3 (2)	C20—C19—H19B	109.2
C4—C3—H3A	119.9	H19A—C19—H19B	107.9
C2—C3—H3A	119.9	C20^iii^—C20—C19	113.8 (3)
C3—C4—C5	120.4 (2)	C20^iii^—C20—H20A	108.8
C3—C4—H4A	119.8	C19—C20—H20A	108.8
C5—C4—H4A	119.8	C20^iii^—C20—H20B	108.8
C4—C5—C6	119.7 (2)	C19—C20—H20B	108.8
C4—C5—H5A	120.1	H20A—C20—H20B	107.7
C6—C5—H5A	120.1	C17—N1—C18	103.10 (19)
C1—C6—C5	119.57 (19)	C17—N1—Zn1	127.13 (15)
C1—C6—C7	118.78 (19)	C18—N1—Zn1	129.41 (17)
C5—C6—C7	121.6 (2)	C17—N2—N3	109.8 (2)
O2—C7—O1	124.67 (19)	C17—N2—C19	129.9 (2)
O2—C7—C6	118.4 (2)	N3—N2—C19	120.3 (2)
O1—C7—C6	116.89 (19)	C18—N3—N2	102.60 (19)
O3—C8—O4	119.1 (2)	C7—O1—Zn2^iv^	119.77 (13)
O3—C8—C2	120.46 (19)	C8—O3—Zn2	86.17 (13)
O4—C8—C2	120.4 (2)	C8—O4—Zn2	94.10 (14)
O3—C8—Zn2	64.09 (13)	C16—O5—Zn2	139.61 (14)
O4—C8—Zn2	55.07 (11)	C16—O6—Zn1	113.53 (14)
C2—C8—Zn2	175.04 (16)	C15—O7—Zn1^v^	104.09 (14)
C10—C9—C14	119.7 (2)	Zn2—O9—H1	126.8
C10—C9—C15	120.7 (2)	Zn2—O9—H2	124.8
C14—C9—C15	119.7 (2)	H1—O9—H2	95.5
C9—C10—C11	120.3 (2)	Zn1—O10—H3	119.4
C9—C10—H10A	119.8	Zn1—O10—H4	121.5
C11—C10—H10A	119.8	H3—O10—H4	106.0
C12—C11—C10	119.8 (2)		

Symmetry codes: (i) *x*, *y*, *z*−1; (ii) *x*−1, *y*, *z*; (iii) −*x*+1, −*y*, −*z*; (iv) *x*+1, *y*, *z*; (v) *x*, *y*, *z*+1.
